# A Pesticide Residues Insight on Honeybees, Bumblebees and Olive Oil after Pesticidal Applications against the Olive Fruit Fly *Bactrocera oleae* (Diptera: Tephritidae)

**DOI:** 10.3390/insects11120855

**Published:** 2020-12-02

**Authors:** Kyriaki Varikou, Konstantinos M. Kasiotis, Eleftheria Bempelou, Electra Manea-Karga, Chris Anagnostopoulos, Angeliki Charalampous, Nikos Garantonakis, Athanasia Birouraki, Fani Hatjina, Kyriaki Machera

**Affiliations:** 1 Department of Entomology, Institute of Olive Tree, Subtropical Crops and Viticulture, ELGO-DIMITRA, Leoforos Karamanli, 73100 Chania, Crete, Greece; n_garantonakis@yahoo.gr (N.G.); abirour@yahoo.gr (A.B.); 2 Laboratory of Pesticides’ Toxicology, Department of Pesticides Control and Phytopharmacy, Benaki Phytopathological Institute, 8 St. Delta str., GR-14561 Kifissia, Greece; E.Manea-Karga@bpi.gr (E.M.-K.); K.Machera@bpi.gr (K.M.); 3 Laboratory of Pesticide Residues, Department of Pesticides Control and Phytopharmacy, Benaki Phytopathological Institute, 8 St. Delta str., GR-14561 Kifissia, Greece; e.bempelou@bpi.gr (E.B.); c.anagnostopoulos@bpi.gr (C.A.); a.charalampous@bpi.gr (A.C.); 4 Department of Apiculture, Institute of Animal Science, ELGO-DIMITRA, 63200 Nea Moudania, Greece; fhatjina@gmail.com

**Keywords:** olive fruit fly, proteinaceous bait sprays, cover sprays, LC-ESI-MS/MS, GC-MS/MS, honeybees, bumblebees, pesticides, honey

## Abstract

**Simple Summary:**

Olive cultivation is extensive throughout the Mediterranean region and essential for the rural economy, local heritage and the environment. To control the olive fruit fly, a major threat for this cultivation, pesticides, in the form of bait or cover sprays, are applied. These pesticide applications can potentially impact pollinators that forage in the nearby areas or live inside the olive orchards. Based on current practice, olive trees supply shadow, water, and still support some flowering plants at the period of the year with the highest temperature and minimum nectar and pollen flow, which can be beneficial for bees. In this study pesticide residues were monitored in honeybees, bumblebees, honey and olive oil, after placement of bee colonies in Greek olive orchards where applications to control the olive fruit fly took place. Variations of concentrations were evidenced, for the three active ingredients that were applied. In limited cases, concentrations in bees higher than the median lethal dose can possibly be attributed to bait dose rates or broad foraging of bees in nearby orchards with similar applications. Determined olive oil residues corroborated that those pesticides were applied in the olive orchards.

**Abstract:**

In 2017 and 2018, a field survey was initiated on Greek olive orchards to investigate the attractiveness of bait spray applications and the impact of cover and bait sprays applied against the olive fruit fly *Bactrocera oleae* (Diptera: Tephritidae), on the honeybee, *Apis mellifera* L. and bumblebees *Bombus terrestris*, by investigating the pesticides’ residual prevalence. Bee colonies were evenly distributed in three sites located on coastal areas of Western Crete and visited almost weekly between July and October. Samples collected, were analyzed using existing or developed-optimized liquid and gas chromatographic methods. In bee samples, concentrations varied from 0.0013 to 2.3 mg/kg for dimethoate, from 0.0013–0.059 mg/kg for its metabolite omethoate, and from 0.0035 to 0.63 mg/kg regarding the pyrethroids, β-cyfluthrin and λ-cyhalothrin. In one bee sample dimethoate concentration exceeded both acute oral and contact median lethal dose (LD_50_). Residue findings in bees, along with verified olive oil residues corroborated that those insecticides had been applied in the olive orchards and transferred to bees. The possibility of non-target effects of the bait sprays to the bees, as well as the impact of the contaminated olive to the bees are discussed.

## 1. Introduction

Olive cultivation is widespread throughout the Mediterranean region and is important for the rural economy, local heritage and the environment. Such cultivation has been practiced since ancient times, with extensive reports on how it was performed, technologies, propagation and practicalities [1]. With an annual production of 300,000 tones, Greece is the third largest producer of olive oil. Crete is the largest island of Greece (South Greece) and 65% of its area is covered by olive groves [2] while there are regions, such as Chania, where olive production as almost a monoculture [3]. Michener reported that the Mediterranean basin is one of the world’s centers of bee speciation and supports some of the most diverse plant-pollinator communities [4,5]. Specifically, the ground vegetation of a typical olive orchard consists of various floral resources that honeybees can rely on during winter and early spring, including nettles, *Urtica* spp. (Urticaceae), *Parietaria officinalis* L. (Urticaceae), annual mercury, *Mercuria lisannua* L. (Euphorbiaceae), common sow thistle, *Sonchus oleraceus* L. (Compositae), common mallow, *Malva sylvestris* L., prickly golden fleece, *Uropermum picroides* (L.), Bermuda buttercup, *Oxalis pes-caprae* L. (Oxalidaceae)) as well as various aromatic plants (rosemary, *Rosmarinus officinalis* L., sage, *Salvia officinalis* L., thyme, *Thymus vulgaris* L., *Cistus incanus* (L.), phrygana (i.e., low scrub habitats with high pollinator diversity and abundance [6,7], ornamental plants (*Lantana* sp. etc.) during the rest of the year.

In Crete, the honeybee colonies are usually close to olive orchards or remain within them during summer, they supply shadow, water, and still support some flowering plants at the period of the year with the highest temperature and minimum nectar and pollen flow. In parallel, olive tree flowers can be a rich pollen source for bees, and recent studies have provided additional verification for this visiting behavior [8].

A typical olive orchard is treated against the olive fruit fly, *Bactrocera oleae, B. oleae* (Rossi) (Diptera: Tephritidae) mainly during summer and autumn season. *B. oleae* is a monophagus insect species with its larvae to be fed exclusively on olive fruits to complete their development. In contrast, the adult mainly female fly feeds on other resources such as pollen, nectar, insect honeydews, and sugar solutions all of which can affect its survival and reproduction.

This behavioral tactic of female olive flies based on their need to search for, and ingest protein for adequate egg production [9] was successfully evaluated by Berlese [10] hence the prevailing name of “Berlese method’’ mean describing the method bait spray. The concept of bait sprays was based on the attraction of the adult female insects by the bait (which are strongly attracted due to ammonia release) and then killed by feeding or come into contact by the sprayed olive stem. Management of the olive fly was based on chemicals and specifically bait or/and cover sprays, which are used almost extensively during the last decade [11,12].

In Greece since 1937, olive orchards were protected from the olive fly attack mainly with bait sprays. Olive growers who were a pivotal agricultural group of Greece, also extensively adopted cover sprays in the olive growing schedule in order to protect their production and increase their income. More specifically, at least two cover sprays with a mixture of two active ingredients were usually applied to the olive agroecosystem to protect olive fruit production from the olive fly damage. Apiculture is also a traditional and well-established occupation in Greece and its interdependence with the agriculture sector (in terms of the need/demand for pollination) highlights its important role. Similarly, bumblebees are also effective pollinators and visitors of a great variety of plants and crops. As bee losses and honeybee mortality incidents are repeatedly reported at national and worldwide level [13,14,15,16,17,18,19,20,21,22,23], oncern was expressed on whether or not the use of fruit flies protein-based baits and cover sprays play a significant role for bees’ survival and normal development. Hence, the association of pesticides with bee health and their accumulation in bees and apiculture commodities needs to be investigated [24,25,26,27], emphasizing the importance of appropriate analytical methods.

Especially for bumblebees, multiresidue pesticides analysis is seldom reported [28,29], considering that principal focus is given to managed honeybees. Nevertheless, last decade’s augmented interest on bee health, exemplified by the European Food Safety Authority (EFSA) guidance document publication [30] and the emphasis given to other pollinators, apart from honeybees, have driven a significant part of research to bumblebees, and solitary bees. Consequently, discussion of the problems and challenges for pesticide residue analysis in bumblebees have been commented by Barganska and colleagues [31].

To this end, the key objective of this study was to evaluate pesticides and selected metabolites’ residues in bee populations positioned inside Mediterranean olive groves for several months. To achieve this goal, multi-residue analysis of pesticides and metabolites was implemented, including extensive analytical method validation for bumblebees. The bee populations included honeybees and bumblebees, and to our knowledge this was the first time that such an integrated survey took place in a Greek olive orchard ecosystem. The monitoring included honey and olive oil produced from olive fruits harvested in the experimental orchard, in an effort to obtain the profile of the specific area regarding the pesticide residues and related contamination.

## 2. Materials and Methods

### 2.1. Experimental Areas

**Figure 1:**
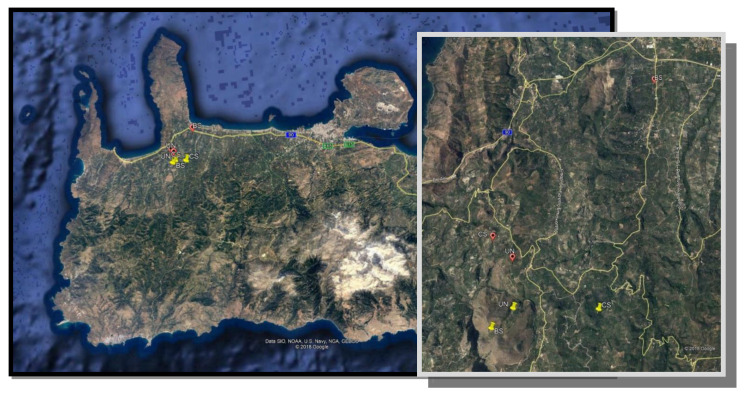
Experimental orchards of Spilia and Astrikas region from where bee samples were taken during 2017 (yellow marks) and 2018 (red marks). CS: orchard treated by cover sprays, BS: orchards treated by Bait sprays and UN: Unsprayed orchard.

To determine the possible effects of protein-based baits and cover sprays on bees, a preliminary field survey was initiated in olive orchards of Chania (Figure 1) during July of 2017 and the whole summer and autumn of 2018. More specifically, large-scale field trials were conducted on the island of Crete in olive groves (cv. Koroneiki with small fruit, the typical oil-producing olive variety planted in Crete) in order to estimate the pesticides’ residual prevalence and potential effects of bait sprays on bees when foraging on olive orchards during summer (under hot and dry conditions whereas no other food simulant (e.g., flower) existed)) and autumn seasons. Three orchards were chosen in a region where olive are cultivated in a is a monoculture and each one was described according to olive fruit fly control methods; Bait sprayed orchard (BS), a cover sprayed orchard (CS) and untreated (UN) served as a control. Each one was at least 750 m away from the other. However, the olive growers who were mainly located in the BS regions high pest pressure and to prevent high olive fruit infestation from the olive fly attack, they apply additionally cover spray applications with insecticides without informing the local authorities in their properties meaning that in BS orchards both methods of bait or cover sprays were mixed (Table 1).

During 2017, the BS orchard was studied in Spilia village (5 ha, 35°31′45.67″ N–23°46′ 39.32″ E_32 m), the CS was in Vasilopoulo (5 ha, 35°29′36.25″ N–23°44′27.77″ E_228 m) while the UN was studied in Vasilopoulo (5 ha, 35°29′21.38″ Β–23°44′39.35″ E_260 m) (Figure 1).

During 2018, the experiment was transferred to Astrikas village with BS (5 ha, 35°28′33.98″ N–23°44′18.75″ E_276 m), CS (5 ha, 35°28′40.66″ N–23°45′48.57″ E_116 m) and UN (5 ha, 35°28′48.24″ N–23°44′37.74″ E_202 m) (Figure 1).

Also, the olive trees at Spilia and Astrikas villages were 50–70 years old, 4–7 m tall and 6–7 m apart (a tree density of 250 trees/ha and they were not irrigated. The olive trees at Vasilopoulo site were not irrigated and were 30–50 years old, 3–6 m tall and 6–7 m apart. The mean olive fruit production per tree was estimated to be approximately 70% and 80% of the normal yield (approximately 80–100 kg/tree) in 2017 and 2018 respectively.

### 2.2. The Insecticide Applications

In bait sprays, a hydrolyzed protein (2%) was mixed with an insecticide in large 1-ton tanks moved by a tractor and applied as a spot treatment (300 mL/tree in the half of the trees of the olive orchard). The liquid hydrolyzed protein was 75% protein (Entomela 75SL, 25% *w*/*w* urea and 75% *w*/*w* protein; Stavrakis, Viotia, Greece). The registered insecticides that were applied in bait spray were β-cyfluthrin (class: pyrethroid; Bulldock 2.5SC; 350 mL/hL; Alfa Georgika Efodia AEBE, Athens, Greece), λ-cyhalothrin (class: pyrethroid; Karate with zeon technology 10 CS; 125 mL/hL; Syngenta Hellas, Athens, Greece), dimethoate (class: organophosphate; Efdakon 40 EC; 625 mL/hL; BASF Hellas, Athens, Greece), and thiacloprid (class: Neonicotinoid; Biscaya 24 OD; 125 mL/hL; Bayer CropScience AG, Athens, Greece), and all of these were mixed with the hydrolyzed protein. Spinosad (class: Naturalytes; Success 0.24 CB; 3.3 L/hL; Dow AgroSciences SA Ltd., Athens, Greece; a ready-made product that includes a new fruit fly bait developed by the company and the toxicant spinosad) was also tested but was not mixed with any of the used hydrolyzed proteins (Table 1). Though in cover sprays, the same active ingredients were also applied alone or combined with others to the whole tree canopy until run off at a dose that is up to ten times lower than that of the bait; the recommended dose for dimethoate is 80–100 mL/hL while for β-cyfluthrin is 15–30 mL/hL. (Table 1).

### 2.3. Timing of the Bait Spray and Cover Spray Applications

Details about the dates, the applied active ingredients and methods are given in Table 1. The number and timing of bait-spray applications in the experimental fields were decided by the Directorate of Agriculture and Veterinary of Chania and were based on the number of McPhail trap catches for each experimental plot and climatic conditions temperature below 28 °C and wind velocity < 5 bf (20–28 km/h or 5.5–7.7 m/s).

Instead the number and timing of cover-spray applications were decided by olive growers and were not based neither in olive fruit fly population nor the infestation; non-systemic pyrethroid ingredients are used to kill the adult fly, systemic organophosphorus to kill the larvae and a combination of them to kill both of them. The first applications (1–2) were applied early in the summer with a pyrethroid and an organophosphorous (OP) pesticide, followed by 1–2 others with an OP.

### 2.4. Sampling of Honey, Honeybees, Bumblebees and Olive Fruits

#### 2.4.1. Sampling of Honeybees, Bumblebees and Honey

Three and five medium honeybee colonies (containing five combs, each with bees) in terms of population and brood were placed in each orchard-type during 2017 (20 June 2017 to end of July) and 2018 (28 June 2018 to end of October) respectively (see Figure S1, for beehives used in this study). The hives were placed on top of a white net of about 1 m^2^. For their maintenance, a beekeeper was visiting them regularly, providing them with 1 kg of sugar candy every month. The colonies had no obvious signs from bee diseases such as *Varroa* mites or *Nosema* microsporidia; no acaricides were applied to the bee colonies while they remained at olive orchards (Figure S1). Similarly, one bumblebee colony was also established in each orchard from early of September to end of October of 2018 (Natupol hives, Koppert, The Netherlands).

Alive adult honeybees and bumblebees were taken before the start of chemical applications and at the day of application. Specifically, 10 individuals were collected weekly from each hive. Each insect was put in a small plastic tube (10 mL) with holes for respiration in its cup and immediately placed in a small cooling box with ice-packs for transportation and soon after they were stored at −80 °C to avoid degradation of materials therein. Honeycombs were collected in the field, and honey was carefully isolated from the honeycomb cells, avoiding contact with cells containing mostly beebread. At the end of the trial the samples were sent (August 2017 and October 2018) at the Laboratory of Pesticides’ Toxicology (BPI, Athens, Greece), where they were stored at −20 °C for the residue analysis. The lag time between storage and analysis did not exceed 3 days.

#### 2.4.2. Sampling of Olive Fruits for Olive Oil Analysis

At the end of the bait and cover spray application against the olive fruit fly (at early harvest period, end of October), samples of olive fruits were collected from all orchards (BS, CS an UN). At least 3 kg of olive fruits/orchard, from minimum 100 trees and 50 fruits/tree (distributed around the whole tree canopy) were collected, in order to achieve a representative sample from the whole tree canopy. Then, samples were immediately transported to the Laboratory of Food Technology of the IOSV and olive oil was extracted at the same day using a laboratory scale olive mill (Callis Company, Athens, Greece). All olive oil samples were packed and sent on the same day to the Laboratory of Pesticide Residues (BPI) for the analysis using gas or liquid chromatography depending on analyte’s physicochemical properties.

### 2.5. Analysis of Samples

#### 2.5.1. Reagents and Standards

Pesticide reference standards (purity > 96%) of all analytes were purchased from Sigma Aldrich (St. Louis, MO, USA), ChemServise (Milan, Italy) and Dr. Ehrenstorfer GmbH (Augsburg, Germany). All solvents, namely acetonitrile (ACN), methanol, acetone, and water were of HPLC-MS grade (Fisher Scientific, Hampton, NH, USA). Graphitized carbon black (GCB), primary secondary amine (PSA) were obtained from Agilent Technologies (Santa Clara, CA, USA). Magnesium sulfate (MgSO_4_) was obtained from Merck (Darmstadt, Germany). Deuterated standards used in the LC-ESI-MS/MS method were imidacloprid-d4, chlorpyrifos-d10, and carbendazim-d3, all obtained from Dr. Ehrenstorfer GmbH. For the acquisition of the data for the rest of the monitored analytes see the previous work [15].

#### 2.5.2. Preparation of Standard Solutions

Analytical standard stock solutions of each pesticide (usually 1000 μg/mL) were made in acetone or ACN and stored at −20 °C (for a maximum period of 3 years). A standard stock solution containing all compounds, at a concentration of 1 mg/L each, was prepared in ACN and also stored at −20 °C. From this solution, working standard solution calibration standards were prepared by the day of the chromatographic analysis of the samples and stored at −20 °C for a maximum of 1 month.

#### 2.5.3. Extraction Procedure

##### Olive Oil

The sample preparation for the detection of pesticide residues in olive oil samples was performed according to previously published method of our group [32]. Five (5 ± 0.1) g of oil sample was extracted with ACN. The sample was then centrifuged at 4000 rpm for 5 min and stored in the freezer (−20 °C) for at least 12 h. Freezing is a critical part of the extraction procedure as it helps to partly remove some additional co-extractives with limited solubility in ACN while the major part of fat and waxes solidify and precipitate. No additional cleanup of fatty components is conducted or required. An aliquot of the still cold ACN phase was transferred into a 15-mL centrifuge tube containing 150 mg of PSA, 12.5 mg GCB and 900 mg of MgSO_4_, the tube was shaken vigorously for 1 min and centrifuged for 5 min at 4000 rpm. Final extracts were analyzed in the chromatographic systems of GC-MS/MS (for GC-MS/MS conditions see Table S1), and GC-ECD for λ-cyhalothrin and β-cyfluthrin and LC-MS/MS for dimethoate (and its metabolite omethoate), thiacloprid and spinosad.

##### Bees and Honey

A modified QuEChERS was implemented for the preparation of the samples. Therefore, sample preparation was based on our recently published work, in which in the second step C18 was utilized in the dispersive mode [14]. The only difference between the GC-EI-QqQ-MS and LC-ESI-QqQ-MS method was in the reconstitution step. Hence, the end extract was divided in two parts: The first aliquot was evaporated under the gentle stream of nitrogen and then readjusted to 1mL (75:25 (*v*/*v*) MeOH: H_2_O solution) for LC-ESI-QqQ-MS analysis. The second aliquot was reconstituted using pure ACN prior to injection to the GC-MS/MS system. Pure ACN was not used in the LC-ESI-QqQ-MS, due to subordinate chromatographic performance of some analytes eluting in the beginning of the chromatogram.

### 2.6. Analytical Method Validation

Both LC and GC methods were validated following the SANTE guidance documents [33,34] on analytical quality control and method validation procedures for pesticide residues analysis in food and feed. Parameters examined were linearity, matrix effect, limit of quantitation (LOQ), specificity, trueness (expressed in terms of recovery percentages), precision, and robustness. Ion ratio and retention time were also evaluated. Bees not exposed to pesticide treatments were used as control samples (blanks), (sample prepared and analyzed before their use for validation purposes). Similarly, blank honey was retrieved from the repository of the laboratory.

More specifically, linearity was assessed by the use of matrix-matched calibration curves in the control bees’ extract and olive oil extract (sample from the market, previously analyzed as to ensure the absence of the analytes under investigation) and standard solutions in solvent. The linearity range tested varied from 1–1000 ng/mL for bees and from 10 to 300 ng/mL (eight concentration levels) for olive oil. The acceptable criterion was a correlation coefficient r > 0.99, accompanied by adequate back-calculated concentrations. Recovery was studied for bees at three concentration levels (LOQ, 10 LOQ, and 100 LOQ) using three experiments per concentration level. As far as olive oil was concerned, recoveries experiments were conducted at two levels (0.010 mg/kg and 0.10 mg/kg) with six replicates at each level. The analysis of pesticide residues in honeybees and honey were conducted following published or ongoing procedures and methodologies of our group [14,15]. For bumblebees although the matrix is similar to honeybees, a thorough validation was performed. In this context, as control bumblebees several individuals from negative (to the analytes monitored) samples of bumblebees (pooled sample from a mortality incident and from the study area) were used for the validation of the method. Lastly, the robustness of the herein presented analytical methods was confirmed after participation in interlaboratory proficiency tests on pesticide residues: (1) in olive oil (COIPT) organized by Italian National Reference Laboratory (Rome, Italy) for pesticide residues and International Olive council, and (2) in honey (PT Scheme 19g—Pesticides—Honeys) organized by the European non-profit organization, Bureau Interprofessionnel d’Etudes Analytiques (BIPEA, Paris, France). To our knowledge, such proficiency test is not available for bees.

### 2.7. Determination of Compounds—Instrumentation

#### 2.7.1. GC-EI-QqQ-MS System and Operating Conditions

The GC-QqQ-MS analysis was performed on a Chromtech Evolution 3 MS/MS triple quadrupole mass spectrometer built on an Agilent 5975 B inert XL EI/CI MSD system. Samples were injected with a MPS-2 autosampler (Gerstel, Mülheim an der Ruhr, Germany) using a 10 μL syringe. Separations were performed on a HP-5 ms UI, length 30 m, ID 0.25 mm, film thick. 0.25 μm (Agilent J&W, Folsom, CA, USA). Helium was used as the carrier gas at a flow rate of 1.2 mL min^−1^. The column oven temperature program started from 50 °C at which was held for 1 min, increased to 125 °C at a rate 25 °C min^−1^, then increased to 300 °C at a rate of 10 °C min^−1^. The triple quadrupole (QqQ) mass spectrometer was operated in EI-MS/MS mode in multiple reaction monitoring (MRM) data acquisition mode. The transfer line, manifold and source of ionization temperatures were 300, 40, and 230 °C. For the MS/MS experiments argon (99.999%) was used as a collision gas and the collision cell pressure was set at 1.7 mTorr. The electron multiplier voltage was set at 2000 V. The total GC analysis time was 36.5 min, employing a solvent delay of 7 min to protect the mass spectrometer filament. GC-MS/MS analysis functioned as a confirmatory medium to GC-ECD findings (see below for respective instrumentation).

#### 2.7.2. LC-ESI-QqQ-MS System and Operating Conditions

The liquid chromatographic (LC) system used was an Agilent1200 Series Quaternary system and chromatographic separation was achieved using an Eclipse XDB C18 column (150 mm, ID 2.1 mm, 3.5 μm) at a flow rate of 0.31 mL/min using a gradient program with a mobile phase consisting of water 5 mmol/L, ammonium formate 0.1%, formic acid 0.02%, and ACN (solvent A) and methanol 5mmol/L, ammonium formate 0.1%, and formic acid (solvent B). Injection volume was 5 μL and in order to avoid carryover, the autosampler was purged with a mixture of methanol/water (50/50 *v*/*v*) before each sample injection. The mass spectrometer used was the Agilent 6410 equipped with an electrospray ionization (ESI) interface operating in the positive mode. Typical source parameters were as follows: capillary voltage (CV) and collision cell energy varied depending on the precursor ion, source temperature was set at 300 °C, drying gas flow rate at 11 L min^−1^, and nebulizing gas pressure at 40 psi. For instrument control, data acquisition, and processing, Agilent MassHunter software version B.01.04 (B84) was used.

Retention time (RT), quantification and qualification ion transitions, CV, and collision energy (CE) for data acquisition in both systems are listed in Table S1. Confirmation of the determination of analytes was based on the criteria of retention time and ion abundance of qualitative and quantitative ions according to the European guideline SANTE/11813/2017 and the method was found to be effective for the extraction of the tested compounds.

#### 2.7.3. GC-ECD Analysis

GC analysis was performed using two Agilent 6890 chromatographic systems connected to ECD detectors, in splitless injector mode. Chromatographic separation was performed on a DB-5-MS column (30 m. 0.32 mm i.d. and 0.25 μm film thickness, Agilent J&W, Folsom, CA, USA) and a DB-17 MS column (30 m. 0.3 mm i.d. and 0.25 μm film thickness, Agilent J&W, Folsom, CA, USA). The use of two different columns was necessary for confirmation purposes. Both systems were under the control of ChemStation chromatography manager and processing software. The helium carrier gas flow rate was set at 1.5 mL/min for both columns. Injectors’ temperature was set at 230 °C and the splitless injection was carried out with the purge valve closed for 1 min. Injection volume was 1 μL. GC-ECD analysis was utilized for pyrethroids detection in bees and honey as well.

## 3. Results

### 3.1. Determination of the Targeted Compounds and Validation of the Analytical Methods

For honeybees and honey, targeted analysis of compounds was performed using already existing methodologies [14,15]. Concerning pyrethroids and LC amenable compounds validation data (not shown) are comparable to the data presented for bumblebees below (and in Table S2 for bumblebees) covering up to 110 active substances. The analytical method proved its efficiency in determining pesticides and metabolites also on bumblebees. More specifically key analytical figures of merit for pyrethroids analysis in bumblebees are presented in Table 2 (similar results were obtained for the analysis with the GC-ECD system). Recoveries varied from 67 to 101% for the three concentration levels tested, with precision values (RSD%) in the range of 6–20%. For rest of compounds monitored by LC-ESI-QqQ-MS see Supplementary Material (Table S2). Concerning linearity, both for GC and LC amenable analytes, it was acceptable with back-calculated concentrations in accordance with the nominal concentrations, accompanied by correlation coefficient values r ≥ 0.99. The sample preparation for bumblebees and honeybees is based on the QuEChERs procedure, but with slight changes compared to other published works concerning bumblebees [28,29]. The use of GCB is not embraced currently by our group for bees; is used only in the olive oil purification step. In the same context, toluene, due to its toxic profile, is not adopted concomitantly with ACN as extraction solvent, despite its efficacy [29].

Similarly, validation results in olive oil samples were in accordance with the EU requirements as being previously presented [32]. Nonetheless, both, for apiculture commodities and olive oil the current work verified that GC-ECD system is still a powerful diagnostic detection system which is in accordance with recent references on its utility in pesticides residues analysis [35,36].

### 3.2. Residues in Honeybees, Bumblebees and Honey Samples

The applications of OPs such as dimethoate and pyrethroids in olive fields constitutes a common practice to control *B. oleae*, the primary insect threat for the olive tree and its principal enemy with severe impact in olive oil production.

The detection of compounds, such as dimethoate, applied in the olive oil orchards, designates a potential risk for the bees. More specifically, dimethoate is a highly toxic compound with an LD_50_ (both acute contact and oral) of 1 mg/kg bee bw [37]. In one case dimethoate’s concentration surpassed the LD_50_ (2.3 mg/kg bee bw, non-treated field), indicating a potential intoxication incident. More interestingly, the finding indicates that adjacent applications might took place, since the sample was collected from a non-treated, with dimethoate, field. In the rest of positive samples, dimethoate, concentrations varied from 0.0018 to 0.65 mg/kg (one sample from the bait field, five samples from cover and one sample from a non-treated field). During 2018 dimethoate samples concentrations displayed lower projection with levels ranging from <LOQ to 0.013 mg/kg (four from a bait field, four from cover and two from a non-treated field).

**Figure 2:**
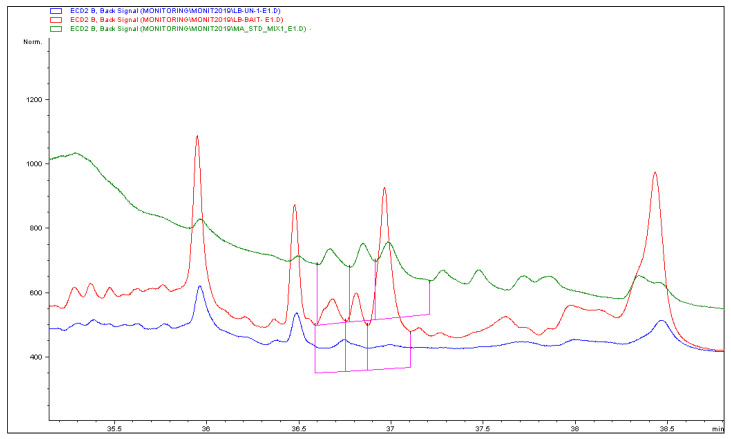
GC-ECD overlayed chromatograms of a honeybee sample positive to β-cyfluthrin (red), a cyflutrhrin matrix matched standard mixture (green) and blank bees extract (blue).

Regarding the analysis for pyrethroids in 2017 samples, only one sample of honeybees was positive to λ-cyhalothrin at 0.14 mg/kg (bait field), a concentration 2.5 times lower than the respective acute contact LD_50_ [38]. The distribution and prevalence of pyrethroids in 2018 was much greater. In this regard, both β-cyfluthrin and λ-cyhalothrin were detected (see Table 3, and Table S3 for analytical results, and Figure 2 for a representative GC-ECD chromatogram). More specifically, β-cyfluthrin demonstrated seven detections in honeybees (4 at bait field, 2 at cover and 1 at non-treated field), while λ-cyhalothrin exhibited six detections (4 at bait field, 1 at cover and 1 at non-treated field).

Concerning bumblebees, only λ-cyhalothrin was quantified slightly above its LOQ (0.0051 mg/kg, at bait field) among all eight samples collected during autumn period.

With regard to honey, the dominant detection concerned dimethoate and its metabolite omethoate. MRLs for dimethoate and omethoate are not established in honey and other apiculture commodities. Therefore, any conclusion is unsafe. Nevertheless, for λ-cyhalothrin the one detection in 2018 (in the bait field), was in proximity to the established MRL (at 0.05 mg/kg). Other active substances (such as thiacloprid and spinosad (Spinosyn A,D)) were not identified in apiculture matrices, with the exception of the acaricide coumaphos, detected (below the LOQ) in two honey samples. The latter was potentially attributed to preexistent contamination of the beeswax (from previous applications).

### 3.3. Analysis of Olive Oil Samples

In olive oil of samples collected during 2017, dimethoate exhibited the higher number of detections (n = 5) with levels varying from 0.01 to 0.64 mg/kg (see Table 4). The residues detected in olive oil produced from olives collected in 2018 (see Table 4), clearly show a preponderance of λ-cyhalotrhin residues, as it was determined in all the experimental orchards of the study, even in samples from an untreated plot which can possibly be attributed to transfer of pesticides droplets via drift phenomena associated with farming practices and sprayer settings-condition as well [39,40,41]. More specifically, Greece is characterized by fragmented land, and the wind transfer of droplets from adjacent applications is possible to occur, and can justify such findings. Unlikely, cross-contamination during oil production in the oil mill cannot be excluded, yet it seldom occurs. β-Cyfluthrin was also determined in samples from bait application, while the insecticides dimethoate, thiacloprid and spinosad applied in the orchards were not detected.

A dissimilar situation was observed in olive oil samples in 2017, as no pyrethroids were detected, whereas dimethoate and thiacloprid were mainly determined. The latter can also be attributed to the far lower oil samples analyzed of this year. Two positive samples for chlorpyrifos were also confirmed, a fact that has to be taken under further elaboration since it is an OP insecticide not authorized for the control of olive fruit fly in olive trees. Nevertheless, it might (as above) pinpoint that chlorpyrifos applications might occurred in nearby cultivations.

## 4. Discussion

The experiments reported herein constitute an exemplary case study examining whether bees are attracted to the fruit flies’ proteinaceous baits and if these insecticides could possibly have a negative effect on bees and other pollinators. In this context, the results showed that during 2018, higher concentrations of dimethoate (and omethoate) were recorded in bumblebees than in honeybees. These concentrations can be assumed less worrying for bumblebees, since the reported LD_50_ values for bumblebees are higher than the ones reported for honeybees (bumblebees: LD_50_ oral >3.3 mg/kg, honeybees: 1 mg/kg) [37]. Nonetheless, bumblebees’ nectar and pollen consumption are higher than that of managed honeybees [30]. Recent reports showed, field realistic doses of insecticides to impact the flight durability of bumblebees [42], which in turn can influence pollination services in key agricultural systems. Consequently, the presented results should be viewed under this perspective as well.

Surprisingly, no mortality of honeybees was observed in 2018, although in one case β-cyfluthrin’s concentration exceeded the LD_50_ endpoint. Though the determined bee body’s burden and the LD_50_ are not directly comparable elements, the fast sampling and the definition of LD_50_, together with the absence of mortality denotes, based on expert judgment, that under field conditions individual bees can survive higher doses than the LD_50_ of β-cyfluthrin. Regarding dimethoate, the concentrations were lower than the LD_50_ values in the majority of the positive findings. Nevertheless, the latter should not be disregarded since OPs are reported to provoke sublethal effects alone or in combination with other active substances. DeGrandi-Hoffmann reported the effects of chlorpyrifos at sublethal level on the queen emergence and virus titers [43]. Dimethoate is also among the active substances that impact the survival and cellular responses of honeybee larvae infected with American foulbrood [44].

Fruit flies use ammonia to locate sources of decaying protein. In contrast, honey bees use visual and olfactory cues to locate flowers and their rewards, namely nectar and their protein source, pollen. The concentrations of various active ingredients, and sometimes the high concentrations detected in bee samples, show that bees were probably attracted to the protein baits and consumed the poisonous substances, which were used in higher dose rates compared to cover sprays; e.g., the dose rate of β-cyfluthrin for bait and cover sprays is 350 and 30 cc/hL, and for dimethoate is 625 and 70 cc/hL, respectively. Similarly, the high residues (0.70 mg/kg) detected in bumblebees can possibly be attributed to the bait dose rate of dimethoate.

Mazor et al. reported that bees preferred between 3 to 10 times more sources of water, which is mixed with NoLure or Buminal proteinaceous baits, than sources of clean water, meaning that bees can also feed in such baits. According to other studies [45] (Dow AgroSciences), a dried product on the leaves is less toxic to honeybees than the wet product. Mangan and Moreno found that the fruit fly attractant component of the bait formulation of *S. spinosa* is repellent to the fruit fly [46]. Recently our group recorded a positive attractiveness of a dried bait spot of α-cypermethrin to bumblebees compared to dimethoate and *S. spinosa* counterpart, as it was indicated by a 54% mortality of bees recorded at the end of the 3-day exposure lab trial [12]. When dimethoate and α-cypermethrin solutions were provided to the bees in liquid form, the recorded mortality was almost doubled (66–94%) while *S. spinosa* solutions were totally harmless to bees. Dimethoate, a broad spectrum insecticide which was often applied by almost all olive growers against all kind of olive pest attacks was also indicated by the residue analysis of this study, however, its registration was ended at the end of June of 2020 based on its high toxicity effects.

Unlike the wide spread effects of conventional cover sprays on beneficial organisms, it has been assumed that protein bait sprays have considerably smaller negative impacts, especially as very low volumes are applied [47,48]. However, it is possible that some other non-target arthropods, apart from bees come into random contact or are attracted to the bait mixtures [49,50]. It has also been hypothesized that a considerable number of insect species including valuable pollinators may be attracted to protein baits [51]. However, very few studies have investigated the attraction of non-target organisms to the protein component of fruit fly baits [50,52] or their capture in traps baited with protein bait [53]. Some studies have reported little or only moderate effects on some non-target organisms. For instance, Thomas and Mangan found no difference in population levels of *Aphytis* sp. before and after the spray of *S. spinosa* bait formulation on Texas citrus. Apart from a few reports showing adverse effects of large scale applications of protein bait sprays on non-target arthropods [54,55,56] no published work has documented the possible negative effects of OPs protein bait spot sprays, on the population dynamics of natural enemies in an orchard cropping system.

The use of honeybee *Apis mellifera* L. (Hymenoptera: Apidae) as a tool for monitoring environmental pollution has been documented in many previous studies [15,57]. Their effectiveness as ecological indicators is based on several behavioural features such as high rate of reproduction, great mobility, large flying range and numerous flower inspections per day it is also based on morphological characteristics: the honeybee body is covered with bristles that collect various particles and by this means increase the contact of the insect with its environment [57]. It has also been postulated that honeybees as insects constitute a better matrix for pesticide and other pollutants detection, especially in terms of magnitude of concentration and number of active substances detected [57,58,59] when compared to honey [60], as honey collected from the colony stores has been already filtered by the bees and it has been stored for at least some weeks before harvested.

The presented results underpin the importance of raising awareness in both olive growers and beekeepers. Especially olive growers should understand that beehives can be in their proximity, and malpractice in plant protection products applications can impact them, and ultimately affect the end products of olive oil and honey. A less important feature not to be neglected, is that bees visit olive trees for pollen, and insect pollination can complement wind pollination, improving the fruit set. Especially for countries in Mediterranean basin, that also possess a strong apiculture sector a framework of actions need to safeguard both pillars. Overall, research studies carried out under realistic field conditions are difficult to give clear cut results due to involvement of many unstable parameters such as the human intervention. Nevertheless outcomes showing bees contaminated with pesticides could sensitize growers to improve their practices. Parallel to this study, other investigations have been already carried out under controlled conditions such as semi-field trials in large cages or a series of lab trials in order to have a clear view concerning the impact of bait and cover sprays on bees/pollinators in Greek olive orchards.

## 5. Conclusions

A battery of gas and liquid chromatographic methods, were used to evaluate pesticide residues in bees, honey and olive oil, after protein bait and cover spray applications in olive orchards in Greece. Findings in bee bodies, along with verified olive oil residues corroborated that those insecticides had been applied in the olive orchards and transferred to bees. In one case the concentration of one organophosphorus insecticide exceeded the acute oral and contact LD_50_, indicating a potential intoxication incident. Concerning bees’ behavior towards protein baits, they were possibly attracted to the protein baits and consumed the respective active substances, which were used in higher dose rates compared to cover sprays. Next steps, include the establishment of semi-field trials or a series of lab trials in order to have a clear view concerning the impact of bait and cover sprays on bees/pollinators in Greek olive orchards.

## Figures and Tables

**Table 1:** Treatments and dates of bait and cover spray applications and possible applied active ingredients from 2017 to 2018 as well as bee sampling dates.

Year Tested	Dates of Bait Sprays	* Applied a.i. + Hydr.pr.	* Dates of Cover Sprays	* Applied a.i.	Sampling Dates
**2017**	14/7	λ-cyhalothrin	11/7	β-cyfluthrin, dimethoate	6/7 13/7 22/7
	28/8	*Saccharopolyspora spinosa (S. spinosa)*	2/8	β-cyfluthrin, dimethoate	N.S.
	22/9	λ-cyhalothrin	11/9	dimethoate	N.S.
	16/10	thiacloprid	1/10	dimethoate	N.S.
**2018**	30/6	β-cyfluthrin	2/7	β-cyfluthrin, dimethoate	28/6 3/7
	10/7	β-cyfluthrin	2/8	β-cyfluthrin, dimethoate	12/7 19/7 26/7
	1/8	dimethoate	3/9	dimethoate	8/8
	21/8	dimethoate		dimethoate	N.S.
	20/9	dimethoate			10/9 24/9 11/10 17/10

* Possible dates of cover with active ingredients (a.i.) and bait sprays with a.i. plus hydrolyzed protein (a.i. +Hydr. pr.) applied by the olive growers. N.S.: No samples were collected.

**Table 2:** Method performance and validation for pyrethroids using GC-EI-QqQ-MS: Limit of Quantitation (LOQ), Recoveries (%), Repeatability (RSD%) and Inter-day (Inter-d) precision (RSD%) obtained in bumblebees.

		Recovery ± RSD%			Inter-d Precision		
		n = 3			RSD%, n = 3		
Analyte	LOQ (mg/kg)	LOQ	10LOQ	100LOQ	LOQ	10LOQ	100LOQ
Deltamethrin	0.005	70 ± 19	70 ± 10	74 ± 6.0	10	11	15
Etofenprox		88 ± 17	82 ± 14	85 ± 10	8	15	17
Tefluthrin		78 ± 19	88 ± 17	95 ± 10	11	15	18
λ-Cyhalothrin		77 ± 11	82 ± 11	81 ± 18	13	17	20
Acrinathrin		80 ± 10	81 ± 17	88 ± 9.2	6	8	15
tau-Fluvalinate		82 ± 3.9	76 ± 7.1	75 ± 8.0	14	11	15
Bifenthrin		85 ± 3.8	79 ± 7.9	82 ± 10	12	8	18
Cypermethrin		80 ± 10	82 ± 15	101 ± 11	6	7	9
Esfenvalerate		82 ± 10	71 ± 5.2	72 ± 5.0	13	13	17
β-Cyfluthrin		85 ± 11	91 ± 12	93 ± 8.6	10	8	14
Permethrin		71 ± 4.7	70 ± 5.3	65 ± 9.2	6	7	8

**Table 3:** Pesticides and concentration ranges in 2017 and 2018 honeybees, bumblebees and honey samples.

**2017**	**Analyte and Concentrations Range (mg/kg)**				**Findings**	
**Matrix**	**Dimethoate**	**Omethoate**	**β-Cyfluthrin**	**λ-Cyhalothrin**	Positive *	**Negative**
Honeybees	0.020–2.300	0.23	nd **	0.14	6	9
Honey	0.0017	nd	nd	nd	1	8
**2018**	**Analyte and Concentrations Range (mg/kg)**					
**Matrix**	**Dimethoate**	**Omethoate**	**β-Cyfluthrin**	**λ-Cyhalothrin**	Positive *	**Negative**
Honeybees	<LOQ–0.013	0.0024–0.020	0.0035–0.630	<LOQ–0.010	24	22
Bumblebees	0.0041–0.700	0.0013–0.059	nd	0.0051	6	5
Honey	0.0057–0.022	0.0051–0.031	nd	0.048	12	15

* to at least one active substance, ** non-detected.

**Table 4:** Pesticides and concentration ranges in 2017 and 2018 olive oil samples.

**2017**	**Analyte and Concentrations Range (mg/kg)**				
**Field Treatment**	**Dimethoate**	**Thiacloprid**	**Chlorpyrifos**	**β-Cyfluthrin**	**λ-Cyhalothrin**
Bait spray	0.014–0.64	0.011–0.028	0.015–0.017	nd *	nd
Cover spray	0.01–0.11	nd	nd	nd	nd
Non-treated	nd	nd	nd	nd	nd
**2018**	**Analyte and Concentrations Range (mg/kg)**				
**Field Treatment**	**Dimethoate**	**Thiacloprid**	**Chlorpyrifos**	**β-Cyfluthrin**	**λ-Cyhalothrin**
Bait spray	nd	nd	nd	0.050–0.056	0.016–0.019
Cover spray	nd	nd	nd	nd	0.010–0.023
Non-treated	nd	nd	nd	nd	0.022–0.024

* non-detected.

## Supplementary Materials

The following are available online at https://www.mdpi.com/2075-4450/11/12/855/s1, Optimization of LC, GC-QqQ-MS conditions, Figure S1: Beehives and the beekeeper who contributed to the study, Table S1: Chromatographic and MRM transition parameters for the GC-EI-QqQ-MS method, Table S2: LC-MS/MS Method performance and validation: Limit of Quantification (LOQ), Recoveries (%), Repeatability (RSD%) and Inter-day (Inter-d) precision (RSD%) obtained in bumblebees, Table S3: Residue analytical results of 2017–2018, Table S4: Residue analytical results in olive oil of 2017–2018.
