# Supplementary material for: A Pesticide Residues Insight on Honeybees, Bumblebees and Olive Oil after Pesticidal Applications against the Olive Fruit Fly Bactrocera oleae (Diptera: Tephritidae)
Source: Insects. 2020 Dec 2;11(12):855. doi: 10.3390/insects11120855 (PMC7760811; doi:10.3390/insects11120855)
Supplement: Supplementary file 1 [file insects-11-00855-s001.pdf]

Supplementary Material

A Pesticide Residues Insight on the Impact of Olive Fruit Fly *Bactrocera oleae* (Diptera: Tephritidae) Spray and Bait applications to Bees.

Kyriaki Varikou[1*], Konstantinos M. Kasiotis[2,*], Eleftheria Bempelou[3], Electra Manea-Karga,[2] Chris Anagnostopoulos[3], Angeliki Charalampous[3], Nikos Garantonakis[1], Athanasia Birouraki[1], Fani Hatjina[4] and Kyriaki Machera[2]

Optimization of LC, GC-QqQ-MS conditions

The MS/MS data acquisition parameters were optimized after injecting pesticide standards and recording their mass spectra in a range from 50 to 800 amu in the electron ionization mode. An abundant mass from the mass spectra of each of the compounds investigated was selected as the precursor ion for the MS/MS fragmentation. Fragmentor voltages varied between 20 to 135 Volts depending on the analyte. After selecting predominant precursor ions, product scans were used to identify main fragments. Once the precursor ions were selected they were subjected to collision energy voltages in order to assess the fragmentation as a result of collision induced dissociation (CID) with nitrogen as the collision gas (argon was the collision gas in the GC-MS/MS). Two multiple reaction monitoring (MRM) transitions (precursor to product ion) at specific fragmentor /collision energies were defined for each target analyte. For each transition in the LC-ESI-MS/MS, the dwell time was set at 15 s, with the exception of dinotefuran set at 50s.

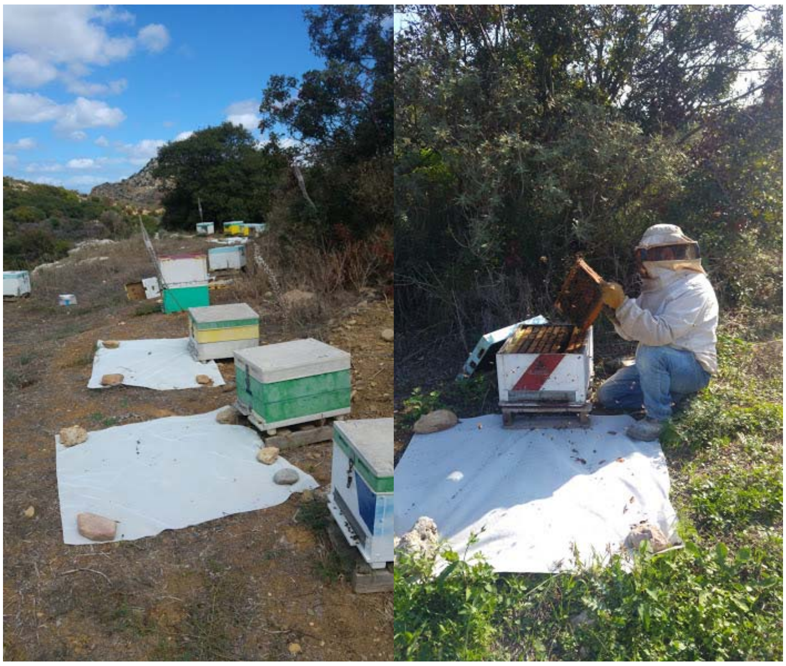

Figure S1. Beehives and the beekeeper who contributed to the study.

Table S1. Chromatographic and MRM transition parameters for the GC-EI-QqQ-MS method.

GC substances	Retention time	Q1 (amu)	Q2 quant (amu)	Collision Energy (eV)	Q1 (amu)	Q2 Identification (amu)	Scan Time (s)	Collision Energy (eV)	MRM transition ratio
deltamethrin	22.09	181	152	20	181	127	0.3	20	18.4
etofenprox	20.71	163	107	9	163	135	0.3	15	47.4
tefluthrin	11.95	177	127	13	177	137	0.3	15	52.6
λ-cyhalothrin	18.78	181	152	15	181	127	0.3	15	28.6
acrinathrin	19.29	181	152	25	181	127	0.3	20	1.9
tau-fluvalinate	21.51	250	55	12	250	200	0.3	12	97.4
bifenthrin	17.79	181	166	10	181	141	0.3	20	8.7
cypermethrin	20.58	181	152	15	181	127	0.3	15	26.6
esfenvalerate	21.56	167	125	15	167	89	0.3	15	3.3
β-cyfluthrin 1	20.58	163	91	15	163	127	0.3	16	31.2
β-cyfluthrin (2,3)	20.75	163	91	15	163	127	0.3	16	31.2
β-cyfluthrin 4	20.92	163	91	15	163	127	0.3	16	31.2
permethrin	19.56	183	153	20	183	168	0.3	20	61.9

Table S2. LC-ESI-QqQ-MS Method performance and validation: Limit of Quantitation (LOQ), Recoveries (%), Repeatability (RSD%) and Inter-day (Inter-d) precision (RSD%) obtained in bumblebees.

Analyte	LOQ (mg/kg)	Recovery ±RSD %			Inter-d precision		
		n = 3			RSD % n = 3		
		LOQ	10 LOQ	100 LOQ	LOQ	10 LOQ	100 LOQ
Acetamiprid	0.001	88±7	85±9	90±14	8	14	16
Azoxystrobin	0.001	72±7	69±6	70±10	10	15	14
Amitraz	0.001	81±11	80±15	78±9	4	5	11
DMF	0.001	88±5	89±7	92±12	11	13	14
DMPF	0.005	73±7	79±10	90±9	7	11	17
DMA	0.01	77±9	79±12	87±9	7	14	14
Benalaxyl	0.005	80±10	74±6	74±12	12	15	18
Cadusaphos	0.001	82±6	79±11	80±8	6	8	7
Diazinon	0.005	102±14	84±8	78±10	7	11	14
Difenoconazole	0.001	71±10	68±8	90±11	8	12	10
Methoxyfenozide	0.001	85±15	78±8	80±10	9	15	18
Thiacloprid	0.001	90±15	84±9	79±13	8	17	20
Triazophos	0.001	82±13	84±15	78±10	10	11	19
Azimsulfuron	0.001	100±14	100±18	95±12	10	8	10
Fenbuconazole	0.001	98±21	85±17	80±12	10	15	16
Fenthion-sulfoxide	0.001	70±15	85±10	83±22	17	15	18
Monolinuron	0.001	99±10	70±17	75±12	8	10	11
Penconazole	0.005	85±4	70±7	75±5	16	12	14
Pendimethalin	0.005	77±4	69±5	67±11	9	10	7
Pirimicarb	0.001	79±10	70±5	72±8	12	11	14
Pirimiphos-methyl	0.001	90±11	78±17	82±10	6	5	14
Prometryn	0.001	78±5	67±10	69±9	5	6	6
Pyraclostrobin	0.001	85±4	80±16	81±8	9	9	15
Spinosyn A	0.001	71±10	74±8	69±8	5	7	8
Spinosyn D	0.001	74±10	80±11	73±10	5	10	12
Spiroxamine	0.001	74±6	68±8	70±10	5	12	18
Tebufenozide	0.005	81±16	82±8	78±8	6	7	8
Thiabendazole	0.001	110±9	98±11	96±17	13	20	19
Trifloxystrobin	0.005	85±18	74±8	73±4	13	10	21
Atrazine	0.005	76±7	75±14	69±8	13	14	10
Boscalid	0.005	69±12	75±10	67±15	12	11	14

Bupirimate	0.005	77±7	72±5	80±8	10	8	20
Buprofezin	0.005	70±6	74±5	82±10	7	9	14
Carbofuran	0.001	72±11	71±14	75±9	5	5	8
Chlorsulfuron	0.005	109±7	85±7	80±7	17	14	11
Coumaphos	0.001	100±12	93±7	91±13	12	20	19
Coumaphos oxon	0.005	93±17	89±15	91±12	8	8	14
Cyprodinil	0.005	89±20	90±18	79±25	10	18	21
Ethion	0.005	71±13	71±10	73±9	10	9	14
Fenamiphos	0.005	80±6	82±5	77±4	13	15	11
Fenamiphos sulfone	0.001	91±12	87±8	90±15	16	17	17
Imazalil	0.005	112±21	91±12	95±13	12	20	21
Iprodione	0.005	75±8	73±10	70±14	13	21	23
Iprovalicarb	0.005	87±8	75±14	77±20	10	14	21
Mepanipyrim	0.005	80±14	82±11	81±15	12	9	22
Metconazole	0.001	89±11	78±12	77±11	15	21	22
Methidathion	0.001	117±20	106±14	105±22	17	14	9
Metoxuron	0.005	80±14	71±18	71±14	7	6	5
Metsulfuron methyl	0.001	94±13	91±12	88±9	8	15	21
Oxadiazon	0.001	67±5	72±6	70±4	10	11	21
Paraoxon-methyl	0.005	65±4	79±13	65±9	15	15	16
Phoxim	0.005	80±8	72±5	82±13	12	21	22
Quinoxyfen	0.005	69±4	60±5	64±3	8	7	14
Tebuconazole	0.005	72±10	70±8	68±12	9	10	13
Thifensulfuron-methyl	0.001	108±21	100±17	97±11	13	17	13
Terbuthylazine	0.005	67±10	68±9	70±11	6	8	7
Zoxamide	0.005	92±9	78±20	80±14	7	14	23
Chlorpyrifos oxon	0.005	77±6	74±5	82±11	10	9	13
Cyproconazole (sum 1+2)	0.005	73±6	71±9	68±9	11	20	22
Diethofencarb	0.005	78±5	69±9	72±10	5	15	12
Fenthion	0.005	75±10	83±11	81±16	13	10	18
Indoxacarb	0.001	78±8	75±6	74±9	8	7	14
Monocrotophos	0.001	69±8	78±10	80±14	5	9	10
Propiconazole	0.005	75±11	60±14	65±21	7	6	14
Terbufos	0.005	85±15	73±15	78±12	8	5	12
Tetraconazole	0.005	81±11	80±15	78±9	4	5	11
Imidaclorpid olefin	0.001	81±11	80±15	78±9	4	5	11

Imidacloprid urea	0.005	82±8	84±15	90±7	13	15	16
Flonicamid	0.005	79±4	71±9	65±21	7	6	14
Bensulfuron-methyl	0.001	63±11	60±11	65±11	15	14	20
Desmetryn	0.001	78±4	70±8	68±11	12	16	19
Fipronil sulfone (ESI$^-$)	0.001	85±10	83±21	82±9	9	9	19
Benfuracarb	0.001	61±12	60±14	64±18	4	7	11
Dimethoate	0.001	90±21	96±17	84±14	17	20	24
Dodemorph	0.001	64±15	58±12	66±11	17	19	24
Ethoprophos	0.001	90±20	78±24	75±9	20	12	25
Metalaxyl M	0.001	79±19	81±12	82±27	22	9	21
Myclobutanil	0.005	92±8	77±8	90±8	25	24	27
Chlorpyrifos ethyl	0.005	94±12	80±12	76±7	20	21	18
Clethodim	0.005	80±8	65±15	72±7	20	21	27
Demeton-S-methyl	0.001	88±6	81±5	78±9	10	12	8
Demeton-S-methyl sulfoxide	0.005	109±10	97±9	95±14	10	12	16
Disulfoton	0.005	92±21	77±20	75±14	14	16	17
Disulfoton sulfoxide	0.005	73±7	76±10	70±8	13	15	17
Fenpropimorph	0.005	82±9	73±9	74±9	19	12	14
Linuron	0.001	85±7	68±9	66±10	19	17	22
Malaoxon	0.001	90±15	82±13	82±17	6	7	12
Piperonil butoxide	0.005	64±11	60±19	63±9	7	12	14
Pyrazophos	0.001	102±8	80±12	81±11	14	10	12
Pyrimethanil	0.001	75±8	65±6	60±9	5	6	8
Thiodicarb	0.001	70±14	68±12	70±8	10	12	14
Tricyclazole	0.001	108±6	94±8	95±18	4	4	12
Acetamiprid N-desmethyl	0.001	74±5	70±7	81±6	10	8	16
Fenpropathrin	0.005	60±11	78±20	71±15	6	5	16
Fenthion-sulfone	0.005	85±20	95±22	97±18	10	8	16
Fipronil (ESI-)	0.001	90±6	95±4	94±5	14	8	25
Malathion	0.005	80±12	70±8	72±15	9	12	24
Phosalone	0.005	71±14	70±27	74±20	21	13	15
Phosmet	0.001	70±15	58±12	60±11	5	14	8
Phosphamidine	0.005	85±10	83±19	85±22	21	13	15
Prothioconazole desthio	0.005	68±7	69±5	73±8	5	4	7
Dinotefuran	0.005	77±7	79±10	88±7	7	9	11
Sulfoxaflor	0.005	77±15	90±12	95±8	12	8	9

Table S3. Residue analytical results in bees and honey (2017-2018).

Field intervention	Sampling	Sampling date	Sample type	Dimethoate (mg/kg)	Omethoate (mg/kg)	β-Cyfluthrin (mg/kg)	λ-Cyhalotrhin (mg/kg)
**Non-treated**	1st Sampling	28/6/18	**Honeybees**	nd	nd	nd	nd
**Bait**			**Honeybees**	nd	nd	nd	nd
**Bait**			**Honeybees**	nd	nd	nd	nd
**Cover-spray**			**Honeybees**	0.0018	nd	nd	nd
**Cover-spray**			**Honeybees**	nd	nd	nd	nd
**Non-treated**			**Honey**	nd	nd	nd	nd
**Bait**			**Honey**	nd	nd	nd	nd
**Cover-spray**			**Honey**	nd	nd	nd	nd
**Non-treated**	2nd Sampling	3/7/18	**Honeybees**	0.0027	0.003	nd	nd
**Bait**			**Honeybees**	nd	nd	nd	nd
**Bait**			**Honeybees**	nd	nd	nd	nd
**Cover-spray**			**Honeybees**	nd	nd	0.009	nd
**Cover-spray**			**Honeybees**	nd	nd	nd	nd
**Non-treated**			**Honey**	nd	nd	nd	nd
**Bait**			**Honey**	nd	nd	nd	nd
**Cover-spray**			**Honey**	nd	nd	nd	nd
**Non-treated**	3rd Sampling	12/7/18	**Honeybees**	nd	nd	0.025	nd
**Bait**			**Honeybees**	nd	nd	nd	nd
**Bait**			**Honeybees**	nd	nd	nd	nd
**Cover-spray**			**Honeybees**	nd	nd	nd	nd
**Cover-spray**			**Honeybees**	nd	nd	nd	nd
**Non-treated**			**Honey**	nd	nd	nd	nd
**Bait**			**Honey**	nd	nd	nd	nd
**Cover-spray**			**Honey**	nd	nd	nd	nd
**Non-treated**	4th Sampling	19/7/18	**Honeybees**	<LOQ	nd	nd	nd

**Bait**			**Honeybees**	nd	nd	15	nd
**Bait**			**Honeybees**	nd	nd	nd	nd
**Cover-spray**			**Honeybees**	nd	nd	nd	nd
**Cover-spray**			**Honeybees**	0.003	nd	nd	nd
**Non-treated**			**Honey**	nd	nd	nd	nd
**Bait**			**Honey**	nd	nd	nd	0.048
**Cover-spray**			**Honey**	nd	nd	nd	nd
**Non-treated**	5th sampling	26/7/2018	**Honeybees**	nd	nd	nd	0.005
**Bait**			**Honeybees**	nd	0.0025	nd	nd
**Bait**			**Honeybees**	nd	nd	nd	nd
**Cover-spray**			**Honeybees**	nd	nd	nd	nd
**Cover-spray**			**Honeybees**	nd	nd	nd	nd
**Non-treated**			**Honey**	nd	nd	nd	nd
**Bait**			**Honey**	0.0089	0.0084	nd	nd
**Cover-spray**			**Honey**	0.013	0.011	nd	nd
**Non-treated**	6th Sampling	8/8/18	**Honeybees**	nd	nd	nd	<LOQ
**Bait**			**Honeybees**	0.013	0.020	nd	nd
**Bait**			**Honeybees**	0.0017	nd	nd	0.005
**Cover-spray**			**Honeybees**	nd	nd	nd	nd
**Cover-spray**			**Honeybees**	nd	nd	nd	<LOQ
**Non-treated**			**Honey**	nd	nd	nd	nd
**Bait**			**Honey**	0.0078	0.0073	nd	nd
**Cover-spray**			**Honey**	nd	nd	nd	nd
**Non-treated**	7th Sampling	10/9/18	**Honeybees**	nd	nd	nd	nd
**Bait**			**Honeybees**	nd	nd	0.036	nd
**Bait**			**Honeybees**	0.0022	nd	0.63	10
**Cover-spray**			**Honeybees**	0.0026	0.0024	0.035	nd
**Cover-spray**			**Honeybees**	nd	nd	0.0035	nd

**Non-treated**			**Honey**	nd	nd	nd	nd
**Bait**			**Honey**	0.021	0.019	nd	nd
**Cover-spray**			**Honey**	0.0057	0.0051	nd	nd
**Non-treated**	8th Sampling	24/9/18	**Honeybees**	nd	nd	nd	nd
**Bait**			**Honeybees**	nd	nd	0.15	<LOQ
**Bait**			**Honeybees**	nd	nd	0.027	nd
**Cover-spray**			**Honeybees**	nd	nd	nd	10
**Cover-spray**			**Honeybees**	nd	nd	nd	0.0042
**Non-treated**			**Honey**	0.018	0.013	nd	nd
**Bait**			**Honey**	0.022	0.012	nd	nd
**Cover-spray**			**Honey**	0.011	0.012	nd	nd
**Non-treated**	8th Sampling	24/9/18	**Bumblebees**	nd	nd	nd	nd
**Bait**			**Bumblebees**	0.70	0.059	nd	nd
**Bait**			**Bumblebees**	0.0043	0.0013	nd	nd
**Cover-spray**			**Bumblebees**	0.0041	0.0017	nd	nd
**Cover-spray**			**Bumblebees**	nd	nd	nd	nd
**Non-treated**	10th Sampling	11/10/18	**Honeybees**	nd	nd	nd	nd
**Bait**			**Honeybees**	nd	nd	226	9
**Cover-spray**			**Honeybees**	nd	nd	nd	<LOQ
**Non-treated**			**Bumblebees**	nd	nd	nd	nd
**Bait**			**Bumblebees**	0.0041	0.0014	nd	nd
**Cover-spray**			**Bumblebees**	0.006	0.002	nd	nd
**Non-treated**		17/10/18	**Honeybees**	nd	nd	nd	nd

**Bait**	11th Sampling		**Honeybees**	0.0017	nd	nd	nd
**Cover-spray**			**Honeybees**	nd	nd	nd	nd
**Non-treated**			**Bumblebees**	nd	nd	nd	nd
**Bait**			**Bumblebees**	0.0045	nd	nd	0.0051
**Cover-spray**			**Bumblebees**	nd	nd	nd	nd
**Non-treated**			**Honey**	nd	nd	nd	nd
**Bait**			**Honey**	0.011	0.0082	nd	nd
**Cover-spray**			**Honey**	0.22	0.31	nd	nd
**Field intervention**	**Sampling**	**Sampling date**	**Sample type**	**Dimethoate (mg/kg)**	**Omethoate (mg/kg)**	**β-Cyfluthrin (mg/kg)**	**λ-Cyhalotrhin (mg/kg)**
**Non-treated**	1st sampling date	6/7/17	**Honeybees**	nd	nd	nd	nd
**Bait**			**Honeybees**	nd	nd	nd	nd
**Bait**			**Honeybees**	nd	nd	nd	nd
**Cover-spray**			**Honeybees**	nd	nd	nd	nd
**Cover-spray**			**Honeybees**	nd	nd	nd	nd
**Non-treated**			**Honey**	nd	nd	nd	nd
**Bait**			**Honey**	nd	nd	nd	nd
**Cover-spray**			**Honey**	nd	nd	nd	nd
**Non-treated**	2nd sampling date	13/7/17	**Honeybees**	0.0023	0.023	nd	nd
**Bait**			**Honeybees**	nd	nd	nd	0.14
**Bait**			**Honeybees**	nd	nd	nd	nd
**Cover-spray**			**Honeybees**	0.65	nd	nd	nd
**Cover-spray**			**Honeybees**	0.010	nd	nd	nd
**Non-treated**			**Honey**	nd	nd	nd	nd
**Bait**			**Honey**	nd	nd	nd	nd
**Cover-spray**			**Honey**	0.0017	nd	nd	nd
**Non-treated**		22/7/17	**Honeybees**	nd	nd	nd	nd

**Bait**	3rd sampling date		**Honeybees**	0.020	nd	nd	nd
**Bait**			**Honeybees**	nd	nd	nd	nd
**Cover-spray**			**Honeybees**	0.59	nd	nd	nd
**Cover-spray**			**Honeybees**	nd	nd	nd	nd
**Non-treated**			**Honey**	nd	nd	nd	nd
**Bait**			**Honey**	nd	nd	nd	nd
**Cover-spray**			**Honey**	nd	nd	nd	nd

nd: non-detected.

Table S4. Residue analytical results in olive oil of 2017-2018.

**Field intervention**	**Sampling date**	**Sample type**	**Dimethoate (mg/kg)**	**Chlorpyrifos (mg/kg)**		**Thiacloprid (mg/kg)**	**β-Cyfluthrin (mg/kg)**	**λ-Cyhalothrin (mg/kg)**
**2017**								
**Non-treated**	31/10/2017	Olive oil	nd	nd		nd	nd	nd
**Non-treated**		Olive oil	nd	nd		nd	nd	nd
**Bait spray**		Olive oil	0.014	nd		nd	nd	nd
**Bait spray**		Olive oil	0.64	nd		0.026	nd	nd
**Bait spray**		Olive oil	nd	0.015		0.028	nd	nd
**Bait spray**		Olive oil	0.017	nd		0.011	nd	nd
**Cover-spray**		Olive oil	0.06	nd		nd	nd	nd
**Cover-spray**		Olive oil	0.01	nd		nd	nd	nd
**Cover-spray**		Olive oil	0.11	nd		nd	nd	nd
**2018**								
**Non-treated**	29/10/2018	Olive oil	nd	nd		nd	nd	0.022
**Non-treated**		Olive oil	nd	nd		nd	nd	0.024
**Non-treated**		Olive oil	nd	nd		nd	nd	0.022
**Non-treated**		Olive oil	nd	nd		nd	nd	nd

**Non-treated**		Olive oil	nd	nd		nd	nd	nd
**Non-treated**		Olive oil	nd	nd		nd	nd	nd
**Non-treated**		Olive oil	nd	nd		nd	nd	nd
**Non-treated**		Olive oil	nd	nd		nd	nd	nd
**Non-treated**		Olive oil	nd	nd		nd	nd	nd
**Bait spray**		Olive oil	nd	nd		nd	0.0523	nd
**Bait spray**		Olive oil	nd	nd		nd	0.0502	nd
**Bait spray**		Olive oil	nd	nd		nd	0.056	nd
**Bait spray**		Olive oil	nd	nd		nd	nd	0.019
**Bait spray**		Olive oil	nd	nd		nd	nd	0.016
**Bait spray**		Olive oil	nd	nd		nd	nd	0.017
**Bait spray**		Olive oil	nd	nd		nd	nd	0.016
**Bait spray**		Olive oil	nd	nd		nd	nd	0.018
**Bait spray**		Olive oil	nd	nd		nd	nd	0.016
**Cover-spray**		Olive oil	nd	nd		nd	nd	0.010
**Cover-spray**		Olive oil	nd	nd		nd	nd	0.011
**Cover-spray**		Olive oil	nd	nd		nd	nd	0.011
**Cover-spray**		Olive oil	nd	nd		nd	nd	0.023
**Cover-spray**		Olive oil	nd	nd		nd	nd	0.022
**Cover-spray**		Olive oil	nd	nd		nd	nd	0.022
**Cover-spray**		Olive oil	nd	nd		nd	nd	0.021
**Cover-spray**		Olive oil	nd	nd		nd	nd	0.023
**Cover-spray**		Olive oil	nd	nd		nd	nd	0.023
